# Dental Education as Seen by a Canada Editor

**Published:** 1878-01

**Authors:** 


### EDITORIAL, ETC.

---

*Dental Education as Seen by a Canada Editor.*—A caustic editorial appears in the November number of the *Canada Journal of Dental Science*, on Dental Colleges, and the "New Departure" of the Western Dental College. The writer thinks that, save and except "the purely operative tuition," he would much prefer the English system of dental curriculum as more solid and disciplinary ; he also thinks that the chief advantages a college offers the student is in epitomizing time, and giving more practical information than can be gotten in a private office. "It is a fact," he writes, "that young men pass through college by the hundred, more filled with conceit than knowledge. * * Graduates themselves over-estimate their advantages." On these grounds the Canada Editor is disposed to think favorably of the plan instituted by the Western College. "It has a chance at least" he says, "of promoting renewed study and application among a large class of qualified men in active practice, who could not attend lectures, but who would be glad of the prospect of getting a degree, to which they might feel that they were entitled by virtue of their knowledge."

He gives some account of the knowledge or rather want of knowledge, on the part of graduates from schools in this country, which would be amusing were it not sad, as showing the utter want of any training, dental or general—showing almost indeed a want of capacity for training.

* * * * "It is a hollow farce," continues the Canada Editor, "to turn out men under existing curriculums with a D. D. S., as if it represented ability. * * There is a serious fault somewhere in the colleges. We believe it is mainly in the curriculum, but it must be partly also in the examinations, and largely too in the want of one or more General Board of Examiners. How many who have passed the examinations in their own colleges, could have succeeded before an impartial central board," * * "Until the bare D. D. S., means more than it does now, it is not worth holding."

Thus vigorously and earnestly writes our Canadian Journalist, with what truth our readers may judge.

This question of Dental Education, we are glad to see, is making a stir, and no better evidence of progress could be afforded than the signs of dissatisfaction with the existing condition of things. Evidently there is room, vast room, for improvement in the methods of teaching and in the things attempted to be taught. But the question of reforming them is one which presents difficulties only seen by those engaged in the teaching, and it seems almost vain to explain these difficulties to those outside, who are so free in their suggestions as to what is needed.

The one great difficulty, as it appears to us, in the way of reform in dental education or dental ethics, is a want of unity and organization among the profession. Representing as it does all grades of intellect and all degrees of cultivation, (we speak of the profession as it stands to-day) there seems not the least harmony, or concert of belief or action on their parts. It is dentist arrayed against dentist in practice, and college arrayed against college in teaching, with what should be a generous rivalry perverted into a tradesman-like competition. Colleges which should confer with each other as to a common method of operations, or a mutual understanding as to the grade of studies and of previous acquirements of students, are kept apart by a spirit which seems to be, if it is not, jealousy.

There is a feature of the case which, while it is humiliating, is worth considering. Supposing, as is most desirable, that a university education were an indispensible pre-requisite to admission to a dental college, as a student; and suppose the course to be at least four years—and this is not only not too long, but hardly long enough to teach a student enough of dentistry to practice profitably to his patients—assuming all this training, will the man who has spent his nine or ten years, with the aims and desires which such training would most likely develop in him,—would such a man be willing to take the place and the pay which nine out of ten of the dentists who now practice are per force com pelled to take? For nine out of ten of those now practising dentistry cannot hope, in the districts in which they are obliged to operate, to find either congenial society or adequate pay. There is no demand for advanced men in most localities, either in medicine or dentistry; they would be dissatisfied, cramped and out of place.

It may seem hazardous to state it, but we feel impelled to the conviction that the average supply to the public of dentists, in point of quality, is equal to the demand. That there are dentists in abundance, as there are M. D's in any quantity, is plain; but the better class of people who patronize dentists do not intelligently discriminate in favor of those who are competent and educated. This is because they do not appreciate the scientific aspect of dentistry. If the public demand for men of education and scientific attainments was increased, the colleges would not be slow to respond.

This demand for additional culture does not come from outside the profession,—for the public, who employ the dentists, though complaining of their work, yet seem incapable in the main of making any adequate distinction or discrimination as to the merits of the respective claimants for practice, nor do they indeed as a rule select as the profession themselves would select;—for the dentist to the dentist is not always the popular operator of the town. The run on a particular office, as is well known, is not due always to the merits of the dentist, and if not fortuitious, is at least not the award of merit.

The pressure,—and it is one of the the things that, as dentists we should feel proudest of,—comes from within the ranks of the profession. The more cultivated are the ones who are urging the elevation of the standard of education, and it is they, not the public, who urge, entreat, demand, reform among those who are by life and act lowering its tone.

Somewhere in the hazy future we see an endowed institution or possibly more than one, selecting with the nicest care and discrimination students with liberal preliminary training,—such training as would admit them to an English University,—and these students so prepared for an ideal dental curriculum, after at least four years' careful training in the principles and practice of dental surgery, graduated. And further on still, we dream of seeing these universities taking the place of and swallowing up the dental colleges, which, having filled a useful place in their day, will become things of the past. But these things cannot come to dentistry sooner than for medicine, nor is it more desirable, and how far off indeed does it seem to be for the last-named profession! Still, the tide sets slowly that way, and the good and wise of the profession are unfeignedly glad to see it. H.

